# Age-prioritized use of antivirals during an influenza pandemic

**DOI:** 10.1186/1471-2334-9-117

**Published:** 2009-07-28

**Authors:** Stefano Merler, Marco Ajelli, Caterina Rizzo

**Affiliations:** 1 Predictive Models for Biomedicine and Environment, Fondazione Bruno Kessler, Trento, Italy; 2 Information Engineering and Computer Science Department, University of Trento, Trento, Italy; 3 National Center for Epidemiology, Surveillance and Health Promotion, Istituto Superiore di Sanità, Roma, Italy

## Abstract

**Background:**

The WHO suggested that governments stockpile, as part of preparations for the next influenza pandemic, sufficient influenza antiviral drugs to treat approximately 25% of their populations. Our aim is two-fold: first, since in many countries the antiviral stockpile is well below this level, we search for suboptimal strategies based on treatment provided only to an age-dependent fraction of cases. Second, since in some countries the stockpile exceeds the suggested minimum level, we search for optimal strategies for post-exposure prophylactic treatment of close contacts of cases.

**Methods:**

We used a stochastic, spatially structured individual-based model, considering explicit transmission in households, schools and workplaces, to simulate the spatiotemporal spread of an influenza pandemic in Italy and to evaluate the efficacy of interventions based on age-prioritized use of antivirals.

**Results:**

Our results show that the antiviral stockpile required for treatment of cases ranges from 10% to 35% of the population for *R*_0 _in 1.4 – 3. No suboptimal strategies, based on treatment provided to an age-dependent fraction of cases, were found able to remarkably reduce both clinical attack rate and antiviral drugs needs, though they can contribute to largely reduce the excess mortality. Treatment of all cases coupled with prophylaxis provided to younger individuals is the only intervention resulting in a significant reduction of the clinical attack rate and requiring a relatively small stockpile of antivirals.

**Conclusion:**

Our results strongly suggest that governments stockpile sufficient influenza antiviral drugs to treat approximately 25% of their populations, under the assumption that *R*_0 _is not much larger than 2. In countries where the number of antiviral stockpiled exceeds the suggested minimum level, providing prophylaxis to younger individuals is an option that could be taken into account in preparedness plans. In countries where the number of antivirals stockpiled is well below 25% of the population, priority should be decided based on age-specific case fatality rates. However, late detection of cases (administration of antivirals 48 hours after the clinical onset of symptoms) dramatically affects the efficacy of both treatment and prophylaxis.

## Background

At the time of writing, a new subtype of influenza A(H1N1) virus is rapidly spreading worldwide [1], with over 39,000 cases and 167 deaths (17 June 2009). Developing measures for controlling the ongoing and future influenza pandemics represents a crucial challenge for public health agencies worldwide.

In order to test the effectiveness of containing/mitigating strategies included in national pandemic preparedness plans, mathematical models have become a relevant tool [2]. Various mathematical models have been proposed for describing the spatiotemporal spread of a possible new influenza pandemic and for evaluating the impact of control measures [3-7].

In general, non-pharmaceutical interventions, such as travel restrictions and social distancing measures, might delay the epidemic arrival and peak, while pharmaceutical interventions, such as the use of vaccines and antivirals, might reduce the overall impact of the epidemic. Specifically, antiviral treatment of influenza cases reduces transmissibility and, according to recent results [8,9], case fatality rates, while post-exposure prophylaxis reduces susceptibility to infection and prevents cases [3].

The World Health Organization suggested that governments stockpile, as part of preparations for the next influenza pandemic, sufficient influenza antiviral drugs to treat approximately 25% of their populations. This recommendation was made with the understanding that the stockpiled drugs would, in the whole, be used for treatment as opposed to significant prophylaxis. Remarkably, however, in many countries the antiviral stockpile is well below the suggested minimum level. For instance, the antivirals stockpiled in Italy are sufficient to treat only 7 million individuals [10], corresponding to the 12% of the population. Therefore, in this study we face the problem of prioritizing the use of antivirals for treatment of cases as a preventive measure for mitigating the spread of an influenza pandemic as long as a pandemic vaccine is not available. On the other hand, in some countries the antiviral stockpile exceeds the number actually required for the treatment of all cases [10]. Thus, we also search for optimal strategies for prioritizing the use of antivirals for post-exposure prophylactic treatment of close contacts of cases. In this case, however, it should be taken into account, that, once the pandemic is well established, antiviral drugs for prophylaxis should also be provided to high-risk healthcare workers and emergency services personnel for the duration of community pandemic outbreaks.

## Methods

To evaluate strategies for prioritizing the use of antivirals in the general population, we performed a systematic simulation study of the spread of an influenza pandemic and of the efficacy of age-prioritized use of antivirals for treatment and prophylaxis, building on the microsimulation model developed for Italy as described in [6,11].

Briefly, the worldwide spread of influenza pandemic and the consequent importation of cases in Italy were modelled using a deterministic homogeneous-mixing SEIR (Susceptible – Exposed, but not yet infectious -Infected – Removed) model. Then we predicted the national impact of the epidemic in Italy using a stochastic, spatially-explicit SEIR model with force of infection depending on the distance and explicit transmission in households, schools and workplaces.

Different transmission scenarios were drawn by varying the basic reproductive number *R*_0 _of the epidemic (i.e. the average number of secondary cases a typical single infected case will cause in a fully susceptible population [12]). In general, the larger the value of *R*_0_, the harder it is to control the epidemic.

Specifically, we considered scenarios characterized by *R*_0 _= 1.4, 1.7, 2. Such values of *R*_0 _comply with those observed in past influenza pandemics [13-16]. Since values of *R*_0 _much larger than 2 were observed in some cities during the 1918–19 Spanish influenza (see [17] for a review), we also considered a scenario characterized by *R*_0 _= 3.

### Transmission models

#### Worldwide transmission model

The worldwide spread of influenza pandemic and the consequent importation of cases in Italy were modelled using a deterministic homogeneous-mixing SEIR model. This model was used for determining the number of imported cases in Italy from abroad over time.

#### National transmission model

**Figure 1:**
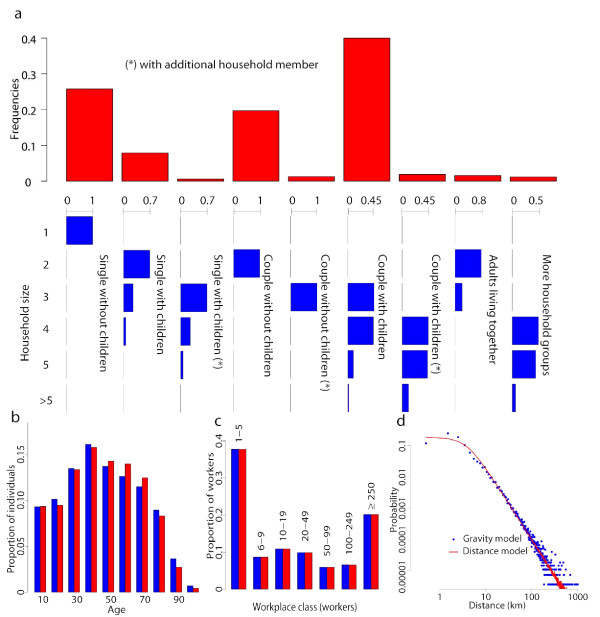
**Sociodemography**. **a **Frequency distributions of household size for the different household types (in blue) and frequency distribution of the different household types (in red) considered in the model. **b **Age distribution from census data (blue) and simulated (red). **c **Proportion of workers for class of workplace from industry census data (blue) and simulated (red). **d **Probability density function of travel distances as obtained by using the gravity model (3) (in blue) compared with that obtained by using the the distance kernel (2) (in red).

The national impact of the epidemic in Italy was predicted using a stochastic, spatially-explicit individual-based model [4,6]. For each individual *i *we define:

• *H*_*i *_as the set of the *n*_*i *_individuals belonging to the same household of individual *i*;

•  as the set of the  individuals attending the same school (index *j *= 1,...,6 identifies school types, from day care centers to university) or sharing the same workplace (index *j *= 7,...,13 identifies workplaces of increasing size, see Figure 1c) of individual *i*;

Any susceptible individual *i*, at any time *t *of the simulation has a probability  of becoming infected, where Δ*t *= 0.25 days is the time-step of the simulation and *λ*_*i *_is the instantaneous risk of infection. The latter is the sum of the risks coming from the three modelled source of infections, namely contacts with infectious members of the household, contacts with infectious individuals working in the same workplace or attending the same school, random contacts in the population:(1)

The terms in Eq. (1) are defined as follows:

• *N *is the total population, i.e. ≈ 57, 000, 000 of individuals;

• *I*_*k *_= 1 if individual *k *is infected, 0 otherwise;

• *β*_*h *_is the within-household transmission coefficient,  are the within-school/workplace transmission coefficients and *β*_*r *_is the transmission coefficient for random contacts. The different transmission scenarios were drawn by varying the transmission parameters.

• *τ*_*i *_is the time in which individual *i *became infectious and *κ*(*T*) is a lognormal function describing infectiousness over time. Estimates of incubation period (1.48 ± 0.47 days) and infectiousness period () lead to a generation time *T*_*g *_= 2.6 days (as in [2]);

• *C*_*k *_= 1 for symptomatic cases (we assume 50% of cases to be symptomatic), 0 otherwise. Since *ω *= 2, the infectiousness of symptomatic cases doubles the one of asymptomatic cases (as in [2]);

• *α *= 0.8 scales the household transmission rates with household size (as in [2]);

•  is a function accounting for induced absenteeism and it is defined as follows: if *T *> 0.25 (the minimum time for recognizing the infection)  is set to: 0.1 for *j *= 1, 2; 0.2 for *j *= 3, 4; 0.25 for *j *= 5; 0.5 for *j *= 6,...,13; 1 otherwise;

• as in [2,4,6], we assume that random contacts in the population depend explicitly on the distance *d*_*ik *_between infectious individual *k *and susceptible individual *i*. The probability that an infectious individual *k *infects individual *i *is weighted by the kernel function(2)

with *a *= 3.8 *km *and *ρ *= 2.32 [6].

We assume that 33% of transmission occurs in households, 33% in schools or workplaces and 33% in the general community [2,6].

### Epidemiological parameters

In the worldwide model, we assumed that infectious individuals were all symptomatic and no longer traveling and that exposed individuals were asymptomatic and possibly traveling before the infectious phase. In the national model, infectious individuals were divided into symptomatic and asymptomatic classes. Once an individual become infectious, the probability of developing symptoms was set to 0.5. In both models, we assumed that the latency period for influenza was the same as the incubation period: duration of 1.5 (± 0.5 standard deviation) days. In the national model, we assumed that the duration of infectiousness varied over time, as a lognormal function [2,4,6]. Infectiousness peaked at 1.75 days, and its duration was truncated at 10 days. This corresponded to an average generation time of 2.6 days. In the worldwide model, the infectious period was assumed to be constant over time and was set at 1.5 days, to give essentially the same growth rate as the national model [4,6].

### Excess mortality

Though it is not possible to predict death rates in future pandemics (reliable estimates are not available yet for the ongoing A(H1N1) influenza outbreak), it is important to assess the effects of antiviral treatment and prophylaxis under different assumptions on age-specific case fatality rates. We used results presented in [18] on the lethal 1918–19 influenza pandemic in Copenhagen (scenario *EM1918*), where deaths occurred primarily among young persons, and in [19] on the mild 1969–70 influenza pandemic in Italy (scenario *EM1969*), where deaths occurred primarily among elderly (as during inter-pandemic seasons), to estimate age-specific case fatality rates. Basically, we assumed that the estimated age-specific excess mortality rates as reported in [18] were associated to an epidemic with *R*_0 _= 2 (authors report estimates of *R*_0 _in 2.2–2.4 for the Summer wave and *R*_0 _≈ 1.2 for the Fall wave, due to preexisting immunity in the population) and we estimated age-specific case fatality rates (for symptomatic individuals) in such a way that the age-specific excess mortality rates as obtained by running simulations with *R*_0 _= 2 comply with the values reported in [18]. The resulting age-specific case fatality rates were used to estimate age-specific excess mortality in all the considered transmission scenarios. Similarly for the data on the 1969–70 influenza pandemic in Italy, where we assumed *R*_0 _= 1.4 (estimated value in the range 1.3–1.6 [20]).

### Imported cases over time

To estimate the number of imported cases over time, we coupled the results of the worldwide model with 2003 data on arrivals and departures in Italy's 38 international airports. More in detail, the number of imported cases over time was estimated by sampling a Poisson distribution of parameter , where *p *is the total number of passengers arriving daily in Italy (≈ 70, 000 on average), *E*(*t*) is the number of exposed individuals at time *t *predicted by the global homogeneous-mixing model, *N *is the world population and Δ*t *is the time step of the simulation.

### Simulated sociodemographic structure

#### Households

In the national model, individuals were randomly grouped in households to match the 2001 census data (Italian Institute of Statistics: XIV Censimento generale della popolazione e delle abitazioni, 2001. Available at url http://dawinci.istat.it/MD/) on age structure and data from a specific 2003 survey (Italian Institute of Statistics: Strutture familiari e opinioni su famiglia e figli, 2003. Available at url http://www.istat.it/dati/catalogo/20060621_03/) on household size and composition. Nine different types of households were considered (e.g., singles or couples, with or without children, with or without additional members, adults living together) and individuals were co-located in households according to specific data on the percentage of the different household types, their size, the age of the household head. Frequency distribution of household sizes for the different household types are shown in Figure 1a, together with the frequency distribution of the household types. The availability of these data allowed us to develop a very realistic model of the mixing of the age classes within households. The resulting age structure of the population is shown Figure 1b and it agrees well with the 2001 census data.

#### Schools and workplaces

The Italian population at 2001 is structured as follows: 20, 559, 595 workers, 11, 360, 556 students and 25, 084, 274 unemployed or retired. Children and young adults were assigned to one of six levels of school (i.e., from day care to university) on the basis of age and specific data on school attendance by age (Italian Ministry of University and Research: La scuola in cifre, 2005. Available at url http://statistica.miur.it/. Italian Ministry of University and Research: L'universitá in cifre, 2005. Available at url http://statistica.miur.it/). Attendance to school varies widely with age: it ranges from 14% in day care centers, to 90% in kindergartens, approximately 100% in primary and middle schools, 82% in high schools, 31% in university. We used specific data on employment rate by age in Italy to assign an employment to individuals aged more than 15 years. Workers were randomly assigned to one of seven employment categories, defined by the number of employees in the workplace (see Figure 1c) (Italian Institute of Statistics: VIII Censimento generale dell'industria e dei servizi, 2001. Available at url http://dawinci.istat.it/cis/). Teachers and school employees were also considered in the model.

#### Commuting

We modelled travel destinations by using specific Italian data on travels between place of residence and place of work or study. Specifically, we used a gravity model [21], in which the probability of commuting from municipality *i *to municipality *j *increases with the population sizes and decreases with the distance:(3)

where *p*_*i *_and *p*_*j *_represent the number of individuals living in municipality *i *and *j *respectively and *d*_*ij *_is the distance between the two municipalities. *θ *is a proportionality constant, *τ*_*d *_= 0.28 and *τ*_*r *_= 0.66 tune the dependence of dispersal on donor and recipient sizes and *ρ *= 2.95 tunes the dependence on the distance. Model parameters were optimized as in [6] in order to take into account that the fraction of commuters (individuals traveling outside the municipality of residence for work or study) in Italy varies significantly from South to North of Italy, ranging from 15% in Southern Italy to 60% in Northern Italy. Figure 1d shows the resulting probability density function of travel distances, compared with that obtained by using a model depending only on the distance, namely Eq. (2), used for modelling the transmission in the general population.

### Prioritizing antiviral treatment and prophylaxis

Both treatment and prophylaxis were assumed to start 24 or 48 hours after the clinical onset of symptoms in the index case. Treatment of the index case was assumed to reduce infectiousness by 70% [2-6], whereas antiviral prophylaxis was assumed to reduce susceptibility to infection by 30%, infectiousness by 70%, and the occurrence of symptomatic disease by 60% [3]. Since it is not realistic that governments will implement prophylaxis without treating index cases first, we consider prophylaxis assuming that antiviral treatment is provided to the index cases. We assumed that 90% of the clinical cases (corresponding to 45% of infected individuals) are identified and treated and that antiviral prophylaxis is provided to the close contacts, namely household contacts, with a treatment course of 10 days [7]. We assumed that treatment with antivirals is associated with a significant reduction in mortality (70%) [8,9].

**Figure 2:**
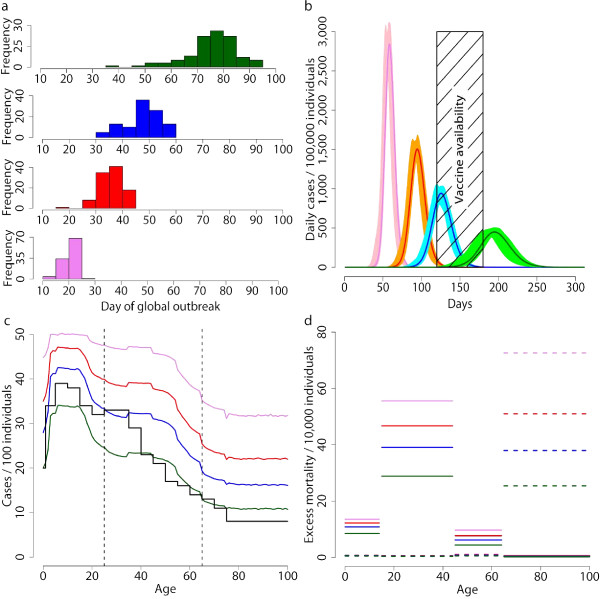
**Baseline simulations**. Timing for initial case for *R*_0 _= 1.4 (green), *R*_0 _= 1.7 (blue), *R*_0 _= 2 (red) and *R*_0 _= 3 (violet) in the baseline scenarios. Histograms are based on 100 simulations each. **b **Expected case incidence over time (solid lines) and 95% confidence intervals (shaded regions) based on 100 simulations for each scenario. Colors as in **a**. The black time window indicate a reasonable time interval for the availability of a pandemic vaccine. **c **Cumulative clinical attack rate by age (colors as in **a**), compared with data on the 1918–19 pandemic [23] (black line). The vertical dashed lines identify the age classes, namely young, adults and elderly, defined for age-prioritization of the use of antivirals. **d **Expected excess mortality by age classes (colors as in **a**) as obtained by assuming two different age-specific case fatality rates, similar to those estimated for the 1918–19 pandemic in Copenhagen (solid lines) and for the 1969–70 pandemic in Italy (dashed lines). Note that in the latter case, the expected excess mortality in the younger age classes (0–64 years old) is very close to 0 for all the *R*_0 _values considered.

We considered administering antiviral treatment and prophylaxis for the entire epidemic period. Population was divided into three classes, namely children and young adults (2–25 years old, individuals younger than 2 years old are excluded since antivirals can not be administered to them [22]), adults (26–64 years old) and elderly (≥65 years old), on the basis of the clinical attack rates by age as resulting from the baseline simulations (Figure 2c), which are consistent with data on attack rates by age classes as reported in [23] for the 1918–19 influenza pandemic. We conducted a systematic simulation study for assessing the effects of targeting the different classes in reducing the number of cases and the excess mortality by minimizing the number of antiviral courses required. To such aim, we consider the number of avoided clinical cases (with respect to the baseline simulations) for each antiviral course as an indicator of efficacy of the different intervention options.

## Results

### Baseline scenarios

**Figure 3:**
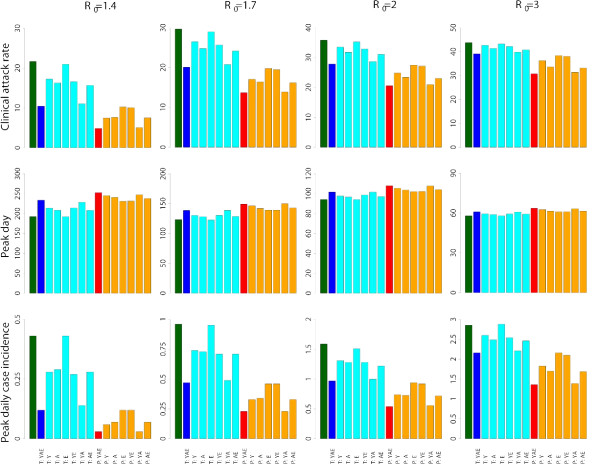
**Age-prioritized use of antivirals during an influenza pandemic in Italy: timing and impact**. Clinical attack rates, peak day and peak daily clinical attack rate for baseline simulations (green), for antiviral treatment provided to index cases of all age classes (blue) or provided only to specific age classes (cyan, Y = young, A = adults, E = elderly), and for post-exposure prophylactic treatment provided to all age classes (red) or only to specific age classes (orange, Y = young, A = adults, E = elderly). When post-exposure prophylactic treatment is considered, we assume that antiviral treatment is also provided to index cases.

On average, the first Italian case arises 76, 48, 36 and 21 days after the first world case for *R*_0 _= 1.4, *R*_0 _= 1.7, *R*_0 _= 2 and *R*_0 _= 3, respectively. Figure 2a shows the stochastic variability in timing of initial case in the baseline scenarios. After the initial highly stochastic phase, the stochasticity decreases over time because of the high number of imported cases over time that, together with long distance travels, contributes to synchronize the local epidemics. Therefore, the simulated epidemics are very stable in terms of parameters as clinical attack rate, peak day and peak daily case incidence. On average, the clinical attack rate is 21.7%, 29.7%, 35.9% and 43.8% for *R*_0 _= 1.4, *R*_0 _= 1.7, *R*_0 _= 2 and *R*_0 _= 3 respectively (see first row of Figure 3). The peak day is at 193, 123, 94 and 58 days respectively (see second row of Figure 3) and the peak daily case incidence is 0.44%, 0.96%, 1.59% and 2.85% respectively (see third row of Figure 3). Figure 2b shows the expected pattern of spread for the different transmission scenarios considered.

**Figure 4:**
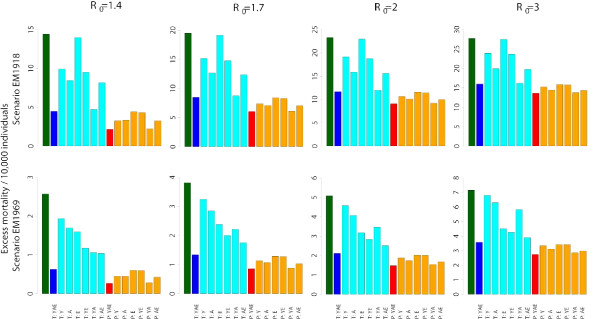
**Age-prioritized use of antivirals during an influenza pandemic in Italy: excess mortality**. Expected excess mortality as obtained by assuming two different age-specific case fatality rates, similar to those estimated for the 1918–19 influenza pandemic in Copenhagen and for the 1969–70 influenza pandemic in Italy respectively, for baseline simulations (green), for antiviral treatment provided to index cases of all age classes (blue) or provided only to specific age classes (cyan, Y = young, A = adults, E = elderly), and for post-exposure prophylactic treatment provided to all age classes (red) or only to specific age classes (orange, Y = young, A = adults, E = elderly).

The time needed from the moment that the vaccine seed virus is available until the first vaccine dose can be used is currently 4 months at best [24]. Other estimates are 6 months at best [25,26]. Remarkably, according to these estimates the pandemic vaccine will be available in time only in case of a mild epidemic (see Figure 2b). Moreover, the continuous importation of cases make unsuitable all containing strategies based on the isolation and treatment of the first clusters of cases. These findings support the hypothesis that, in large countries, social distancing measures (e.g. school and non essential workplaces closure, case isolation), travel restrictions and pharmaceutical measures based on antiviral treatment of index cases and prophylaxis to close contacts will be key in mitigating and delaying the epidemic as long as the pandemic vaccine is not available. Figure 2d shows the expected age-specific excess mortality in the four considered transmission scenarios and by assuming two different patterns of mortality, namely scenarios *EM1918 *and *EM1969*. In the *EM1918 *scenario, the excess mortality is estimated to be 14.4*/*10, 000, 19.5*/*10, 000, 23.3*/*10, 000 and 27.8*/*10, 000 for *R*_0 _= 1.4, *R*_0 _= 1.7, *R*_0 _= 2 and *R*_0 _= 3 respectively (see first row of Figure 4). In the *EM1969 *scenario, it is estimated to be 2.6*/*10, 000, 3.8*/*10, 000, 5.1*/*10, 000 and 7.2*/*10, 000 respectively (see second row of Figure 4).

### Age-prioritized use of antivirals: early detection of index cases

**Figure 5:**
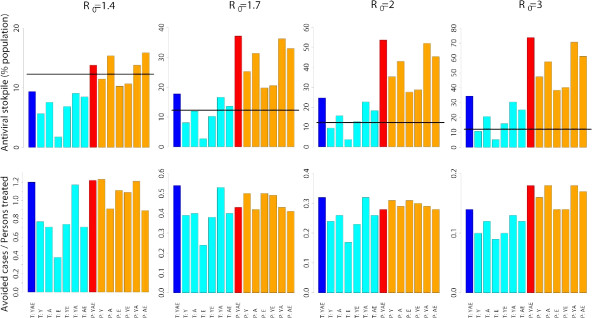
**Age-prioritized use of antivirals during an influenza pandemic in Italy: antiviral stockpile**. Antiviral stockpile required and number of avoided cases divided by the number of persons treated for antiviral treatment provided to index cases of all age classes (blue) or provided only to specific age classes (cyan, Y = young, A = adults, E = elderly), and for post-exposure prophylactic treatment provided to all age classes (red) or only to specific age classes (orange, Y = young, A = adults, E = elderly). When post-exposure prophylactic treatment is considered, we assume that antiviral treatment is also provided to index cases. The horizontal black line represents the Italian antiviral stockpile.

We first assume that index cases and close contacts are treated 24 hours after the onset of symptoms in the index cases. If antivirals are used for treatment only, for all age classes, attack rates will decrease to 10.5%, 20.1%, 27.9% and 39.1% (see first row of Figure 3), requiring an antiviral stockpile for treating 5, 10, 14 and 20 million individuals (corresponding to the 9.4%, 17.8%, 24.7% and 34.6% of the population, see first row of Figure 5), for *R*_0 _= 1.4, *R*_0 _= 1.7, *R*_0 _= 2 and *R*_0 _= 3 respectively. Moreover, the epidemic peak is slightly delayed (of about 37, 15, 8 and 3 days respectively, see second row of Figure 3) and the peak daily case incidence is greatly reduced (by about 72%, 51%, 39% and 24% respectively, see third row of Figure 3). The number of avoided cases for each antiviral course is 1.2, 0.54, 0.32 and 0.14 respectively (see second row of Figure 5). The excess mortality is greatly reduced by assuming age-specific case fatality rates as those estimated for both the 1918–19 and 1969–70 pandemics. In the *EM1918 *scenario, the excess mortality is reduced by 69%, 56%, 50% and 42% for *R*_0 _= 1.4, *R*_0 _= 1.7, *R*_0 _= 2 and *R*_0 _= 3 respectively (see first row of Figure 4). In the *EM1969 *scenario, the excess mortality is reduced by 75%, 65%, 58% and 50% respectively (see second row of Figure 4).

Treatment of elderly does not lead to any significant reduction of the cumulative number of cases, while the effects of treating younger population and adults are similar to those observed when treatment is considered for all age classes. This means that treatment of elderly has a poor effect in reducing the cumulative attack rate. In fact, when treating only the elderly, the number of avoided cases for each antiviral course decreases by 68%, 56%, 47% and 36% for *R*_0 _= 1.4, *R*_0 _= 1.7, *R*_0 _= 2 and *R*_0 _= 3 respectively. The number of avoided cases for each antiviral course is similar when treatment is considered only for young or adult individuals (see last row of Figure 5).

Results can be different when considering the effects on excess mortality (see Figure 4). By assuming age-specific case fatality rates similar to those estimated for the 1918–19 influenza pandemic in Copenhagen, treatment of adults is much more effective (the excess mortality decreases by 41.2%-28% for *R*_0 _in 1.4–3) than treatment of elderly population (the excess mortality decreases only by 2.8%-0.8% for *R*0 in 1.4–3). The opposite pattern is observed by assuming age-specific case fatality rates similar to those estimated for the 1969–70 influenza pandemic in Italy: treatment of adults is much less effective (the excess mortality decreases by 33.8%-12% for *R*_0 _in 1.4–3) than treatment of elderly (the excess mortality decreases by 37.9%-36.9% for *R*_0 _in 1.4–3).

When prophylaxis is provided to close contacts of index cases, the clinical attack rates decrease to 4.9%, 13.7%, 20.6% and 30.8% (see first row of Figure 3), but a larger antiviral stockpile is required (sufficient to treat 8, 21, 31 and 42 million individuals, corresponding to the 13.8%, 37.2%, 53.8% and 73.8% of the population, see first row of Figure 5), for *R*_0 _= 1.4, *R*_0 _= 1.7, *R*_0 _= 2 and *R*_0 _= 3 respectively. Moreover, the epidemic peak is significantly delayed (of about 60, 26, 14 and 6 days respectively, see second row of Figure 3) and the peak daily case incidence decreases (approximately by 93%, 76%, 66% and 52% respectively, see third row of Figure 3), with respect to the baseline scenarios. The number of avoided cases for each antiviral course is similar to that observed for antiviral treatment, namely 1.22, 0.43, 0.28 and 0.18, respectively for the four transmission scenarios considered (see second row of Figure 5). By assuming age-specific case fatality rates similar to those estimated for the 1918–19 influenza pandemic in Copenhagen, the excess mortality decreases by 51.6%, 29%, 22.1%, and 14.7% with respect to treatment of all cases for *R*_0 _= 1.4, *R*_0 _= 1.7 *R*_0 _= 2 and *R*_0 _= 3 respectively (see first row of Figure 4). The excess mortality decreases even more by assuming age-specific case fatality rates similar to those estimated for the 1969–70 influenza pandemic in Italy, namely 56.5%, 36%, 29.7%, and 23.2% (see second row of Figure 4). Providing prophylaxis only to individuals in some age classes results in the same patterns observed above for the age-prioritized treatment of index cases (see Figure 3 and Figure 5). Age-prioritized prophylaxis does not result in a significant reduction of the excess mortality with respect to the treatment of all cases (see Figure 4). In fact, when considering prophylaxis to close contacts of cases we are assuming that treatment is first provided to all index cases.

Prophylaxis provided to younger individuals is the only intervention allowing a relevant reduction of the cumulative clinical attack rates (they decrease to 7.5%, 17.1%, 24.9% and 36.3%, respectively for the four transmission scenarios considered) with a significant reduction of the antiviral stockpile required (sufficient to treat 7, 14, 20 and 27 million individuals, corresponding to the 11.5%, 25.3%, 35.3% and 47.5% of the population), at least when *R*_0 _is no much larger than 2 (see Figure 3 and 5).

### Age-prioritized use of antivirals: late detection of index cases

We now assume that index cases and close contacts are treated 48 hours after the onset of symptoms in the index cases. It is worth noticing that this delay results in a dramatic decrease of the intervention efficacy and, in general, a larger number of antivirals stockpiled is required and a lower decrease of the clinical attack rate is observed. When treatment is considered for all index cases, the clinical attack rate decreases to 14.9%, 23.9% and 31.1% for *R*_0 _= 1.4, *R*_0 _= 1.7 and *R*_0 _= 2 respectively and the number of avoided cases divided by the number of persons treated decreases to 0.51, 0.27 and 0.18. When prophylaxis is also considered, the clinical attack rate decreases to 9.1%, 17.9% and 25.1% for *R*_0 _= 1.4, *R*_0 _= 1.7 and *R*_0 _= 2 respectively and the number of avoided cases divided by the number of persons treated decreases to 0.55, 0.27 and 0.19. Even worst efficacies are observed when *R*_0 _= 3.

### Realizations and results variability

Results presented in this section were obtained by averaging over 15 simulations for each transmission scenario considered (but for the baseline simulations which were based on 100 simulations). This certainly represents a number large enough to guarantee the stability of the results. Specifically, only the timing of the initial cases is highly variable (however, this is due to the high stochasticity of the epidemic in its initial phase). On the contrary, the epidemiological indicators depending on the whole course of the epidemic are very stable: standard deviations are less than 0.02% of the population for the cumulative attack rates, less than 6 days for the peak day and less than 0.04% of the population for the peak daily case incidence.

## Discussion

A recent study conducted in Italy [6] has shown that the use of antivirals, for treatment of index cases and post-exposure prophylactic treatment of household contacts, is the most effective single intervention strategy, resulting in a relevant reduction of the cumulative clinical attack rate, namely of 78%, 50% and 36%, for *R*_0 _= 1.4 *R*_0 _= 1.7 and *R*_0 _= 2.1, respectively. In addition, their use contributes to delay the epidemic peak and to decrease the peak daily case incidence. Similar results have been shown for UK and US [4,5]. Moreover, school and workplace prophylaxis could dramatically increase the impact, in terms of reduction of the clinical attack rate [4,6].

However, critically, the antiviral stockpile required is relevant. In Italy [6], an antiviral stockpile large enough to treat 20 to 30 million of individuals (corresponding to the 35% and 53% of the population) is needed for *R*_0 _in 1.7 – 2. For *R*_0 _= 1.7, the antiviral stockpile required decreases to 17% of the population only with the availability of a vaccine within 4 months after the first world case and by considering large scale social distancing measures (e.g., 90% air travel restriction and school closure for 2 months). For *R*_0 _= 2.1, the antiviral stockpile required is about 35% of the population. Similar results were obtained in US [4], where the antiviral stockpile required ranges from 25% to 60% of the population, depending on the transmission scenario and the different mitigation measures considered (school/workplace prophylaxis excluded).

We conducted a systematic simulation study of the age-prioritized use of antivirals for mitigating and delaying an influenza pandemic. By assuming *R*_0 _no much larger than 2, our results confirm that the antiviral stockpile required for the treatment of cases ranges from 10% to 25% on the basis of the transmission scenario considered. If *R*_0 _= 3, the stockpile required for the treatment of cases increases to 35% of the population. Treatment of index cases is effective in mitigating the epidemic (decrease of cumulative attack rate ranges from 11% to 52% in the four considered transmission scenarios, decrease of peak daily case incidence ranges from 24% to 72%). By assuming that treatment with antivirals is associated with a significant reduction in mortality (70%), a large decreases in the excess mortality is observed in all the transmission scenarios considered (ranging from 42% to 75%).

No suboptimal strategies, based on the treatment of a fraction of cases on an age basis, were found able to remarkably reduce both the clinical attack rate and the antiviral stockpile required. Remarkably, however, a significant reduction of the excess mortality can be achieved by treating only a specific fraction of the population, depending on age-specific case fatality rates: treatment of adults is more effective if age-specific case fatality rates are similar to those estimated for the 1918–19 influenza pandemic in Copenhagen while treatment of elderly is more effective if age-specific case fatality rates are similar to those estimated for the 1969–70 influenza pandemic in Italy. Therefore, early estimates of age-specific cases fatality rates can be crucial for optimizing the use of antivirals during an influenza pandemic. Moreover, we have shown that treatment of elderly does not lead to any significant reduction of the cumulative attack rate and that the efficacy of treating younger population and adults are similar, but with a different cost in terms of antiviral doses required.

Treatment provided to all cases coupled to prophylaxis for younger individuals is the only intervention allowing a significant reduction of the cumulative clinical attack rate with a significant reduction of antiviral courses required, with respect to provide prophylaxis to the all close contacts of cases. To implement this strategy, the antiviral stockpile should be large enough to treat about the 12%, 25%, 35% and 47.% of the population, for *R*_0 _= 1.4, *R*_0 _= 1.7, *R*_0 _= 2 and *R*_0 _= 3 respectively. Since the antivirals stockpiled in Italy are sufficient to treat only about 7 millions individuals, corresponding to the 12% of the population, Italy seems to be able to mitigate an influenza pandemic only at the very beginning of the outbreak. However, the implementation of social distancing measures (e.g. isolation of index cases and school/workplace closure), travel restrictions could slow down the spread of the epidemic. Consequently, the antiviral stockpile required could be significantly lower than that predicted by our model and time could be gained for pandemic vaccine production and distribution, at least under moderate transmission scenarios.

In our study we did not consider treatment and prophylaxis for specific categories, such as patients admitted to hospital, health care workers with direct patient contact and emergency medical service providers, highest risk patients (young children 12–23 months old, elderly ≥65 years old), public safety workers (police, fire, corrections), and government decision makers. These policies are consistent with medical practice and ethics to treat those with serious illness and who are most likely to die and those groups which are critical for an effective public health response to a pandemic (preventing absenteeism and maintaining societal functions). Specific work should be conducted for modelling these interventions in order to refine our estimates. However, our strategies of age prioritization could have important ethical impacts that should be taken into account. Recently, the WHO has developed specific guidelines to take into account ethical considerations in developing a public health response to pandemic influenza [27]. As regards age-based prioritization, it is stated that "the goal of reducing overall disease burden might also provide a rationale for favouring younger persons, even if the fair innings argument is not accepted".

However, "age-based prioritization criteria should be adopted only after wide public consultation". Moreover, the potential impact of resistance of the circulating strain to antiviral drugs should also be considered [28-31]. The extent of such may cause substantial revision to polices regarding the use of such drugs during the next pandemic. In fact, our results should also consider the possibility of the emergence of an antiviral resistant strain as observed in the last two influenza seasons for influenza A(H1N1) strain [32]. The circulation of transmissible oseltamivir-resistant virus may preclude the use of oseltamivir for post-exposure prophylactic treatment of close contacts. However, certain countries have differentiated their stockpile acquiring also zanamivir which is particularly relevant in light of emerging resistance to oseltamivir. This implies that additional antiviral reserve capacity is required and this is likely to come primarily from zanamivir [32].

Our study also highlights the importance of the early detection of cases. In fact, great effort should be made in order to establish a surveillance system able to detect and treat cases as soon as possible since a delay of more than 24 hours could make both antiviral treatment and prophylaxis very inefficient. This means that, to be successful, preparedness to pandemic should not be only stockpiling of antiviral courses. A great effort should also be made in organizing antiviral distribution and implementing specificity and sensitivity of existing surveillance systems for seasonal influenza, in order to detect cases as soon as possible in the occurrence of the emergence of an influenza pandemic. Antiviral drugs must be given early in the course of infection to reduce symptoms (maximum 48 hours) and before any prospect of knowing the sensitivity of the virus [33,34]. Viral loads begin to decrease 24–48 hours after he onset of symptoms and late antiviral therapy is unhelpful [35]. This critical aspect may have important implications on infrastructure for care delivery. Since health systems may be overwhelmed during a pandemic, new care services for providing the usual health care services (such as drug delivery in hospital or in pharmacies or directly at home) should be considered in order to timely distribute antivirals to cases and close contacts. Also, monitoring systems able to detect adverse events should be considered. However, this aspects are directly related to the organization of the health care system, and should be tailored on the basis of the different resources available.

A characteristic feature of pandemics is to appear in a series of waves. Results presented in this work could be considered fairly unrealistic if waves were determined by virus mutations resulting in the elimination (even partial) of acquired immunity in the population. In fact, a much larger cumulative attack rate would be expected during a series of wave in which acquired immunity is lost at the end of each wave. On the contrary, no substantial differences, but for the timing of the epidemic spread, would be expected if waves were determined by factors that do not contribute to increase the effective reproductive number (e.g. school closure in the Summer period or spontaneous behavioural changes of the population in response to the epidemic [36]).

## Conclusion

Our results strongly suggest that governments stockpile sufficient influenza antiviral drugs to treat approximately 25% of their populations, by assuming that *R*_0 _is not much larger than 2. In fact, no suboptimal strategies, based on the treatment of a fraction of cases on an age basis, were found able to reduce remarkably both the clinical attack rate and the antiviral stockpile required. In countries where the number of antivirals stockpiled is well below 25% of the population, treatment of elderly should be considered as a priority if age-specific case fatality rate were similar to that estimated for the 1969–70 influenza pandemic in Italy, where deaths occurred primarily among elder persons. On the contrary, treatment of adults should be considered as a priority if age-specific case fatality rate were similar to that estimated for the 1918–19 influenza pandemic in Copenhagen, where deaths occurred primarily among adult persons. In countries where the number of antiviral stockpiled exceeds the number required for the treatment of cases, providing prophylaxis only to younger individuals is an option that could be taken into account in the preparedness plans. However, these results are influenced by the timing of cases detection: administration of antivirals 48 hours after the clinical onset of symptoms in the index cases dramatically affects the efficacy of both treatment and prophylaxis.

## Competing interests

The authors declare that they have no competing interests.

## Authors' contributions

SM and MA developed the model. SM and CR conceived and designed the experiments. SM and MA performed the experiments. SM, MA and CR contributed to interpreting the results. SM wrote the paper. All the authors have read and approved the final version of this paper.

## Pre-publication history

The pre-publication history for this paper can be accessed here:

http://www.biomedcentral.com/1471-2334/9/117/prepub
